# CMOS Capacitive Fingerprint Sensor Based on Differential Sensing Circuit with Noise Cancellation

**DOI:** 10.3390/s18072200

**Published:** 2018-07-08

**Authors:** Hossam Hassan, Hyung-Won Kim

**Affiliations:** 1 Department of Electronic Engineering, College of Electrical and Computer Engineering, Chungbuk National University, Cheongju 28644, Korea; 2 Department of Electronics, National Telecommunication Institute, Nasr City, Cairo 11768, Egypt

**Keywords:** charge sharing detection, differential integrator, charge integration, capacitive-sensing, capacitive fingerprint sensor

## Abstract

In this paper, we introduce a differential sensing technique for CMOS capacitive fingerprint detection. It employs a new capacitive-sensing cell structure with charge sharing detection and readout circuit. The proposed technique also can eliminate the effect of parasitic capacitances by employing parasitic insensitive switched-capacitor structure and so increases the sensitivity even under severe noisy conditions. It can also overcome the performance degradation caused by various conditions of finger surface by using a differential integrator and adjusting its number of integrations. In addition, the proposed architecture allows parallel detection of all sensing channels. It can, therefore, substantially speed up the detection process compared with conventional architectures. We implemented a prototype fingerprint sensor chip with an array of 20 × 16 sensor cells using a 130 nm CMOS process. Simulation experiments demonstrated that the proposed architecture provided an SNR gain of 54 dB, whereas a conventional single line sensing gives an SNR gain of only 13 dB.

## 1. Introduction

Nowadays, the increasing requirements on tighter security are raising the demand of the accuracy and performance levels of biometric sensing techniques such as fingerprint sensing. User authentication becomes one of the major challenges in the era of mobile banking, FinTech, and the Internet of Things (IoT). By using traditional biometric technologies combined with context-aware authentication techniques, researchers have proposed the Internet of Biometric Things (IoBT) [1,2,3]. Among a variety of biometric techniques, fingerprint recognition is one of the most popular biometric techniques in the current market. For example, recent smartphones commonly adopt fingerprint sensors for a biometric identification method [4,5,6]. The key property of fingerprints is that no two individuals have the same fingerprints and aging does not affect the fingerprints [5].

There are many fingerprint sensing methods such as optical methods [7,8], thermal methods [9], ultrasonic methods [10], RF imaging methods [6] and capacitive methods [11,12,13,14]. The capacitive method is the preferred solution for mobile and IoT applications due to the compact size and low power consumption of capacitive fingerprint sensor chips. Small and low-cost capacitive fingerprint sensors have been presented in References [13,14,15]. Most of the previous works, however, suffer from the problem of unstable sensitivity under variable finger conditions (wet finger and dry finger) and noisy environment, which significantly degrades the image quality. Several ideas have been proposed to enhance the image quality by using a local threshold level or by adding compensation capacitors [16], or using the voltage drop suppression [11], or applying an effective isolation structure [12].

Recently, many research groups have been working towards embedding the fingerprint sensor in-cell phone display. On the other hand, there are other research groups work for the development and improvement of the stand-alone fingerprint sensing. The in-cell phones display fingerprint sensors give the user more space and more comfort. The stand-alone fingerprint sensor can be embedded out of the cell phones display and in non-cell phone devices. For fingerprint sensor in-cell phones display, there are three competitive solutions and prototypes that include the fingerprint sensor.

The first prototype uses the ultrasonic method [10,17] and it is introduced to cell phones by Qualcomm Technologies, Inc. [18,19,20,21]. It uses ultrasonic waves to penetrate the internal structure of the finger and map the dermal structure of the fingerprint. It can be added under the display of the cell phone to capture the fingerprint image and it can image through metal and opaque glass or plastic layers. Tang et al. presented a fully integrated 3-D ultrasonic fingerprint sensor-on-a-chip [10,17]. The ultrasonic method needs a special device—array of piezoelectric micromachined ultrasonic transducer (PMUT)—on top of the CMOS device with a 24 V driving signal. For mass production of the ultrasonic fingerprint sensors for the in-cell phones display fingerprint, it needs complex manufacturing steps and its fingerprint recognition speed is slow [5,17]. Also, it needs to achieve a high performance at a lower or similar cost and power consumption of the capacitive technology.

The second prototype uses an optical method [22], and it is introduced to cell phones by Synaptics, Inc. [23,24,25,26]. The optical fingerprint sensor is added under the display of the cell phone to capture the fingerprint image where a special position of the display works as a fingerprint scanner. This prototype is embedded under the display; however, it has the drawbacks of working under the strong light and dealing with certain circumstances such as dry fingers and wet fingers. Moreover, they cannot get 3-D data from below the finger surface and in general optical technology may be easier to spoof than others such as ultrasonic and capacitive methods. 

The third prototype is the capacitive method [27,28,29] which uses on-display mutual capacitance to provide a fingerprint sensing functionality. This prototype [28,29] verified a 500-dpi transparent on-glass fingerprint sensor using 15-V TX driver. The capacitive sensing method uses the projective capacitance technique used for the touchscreen to implement the fingerprint sensing for the in-cell phone display [28,29]. The drawbacks of this approach like that of the touchscreen such as the need for a high TX voltage around 15–20 V to compete with the panel noise and surrounding noise [28,29,30,31].

Although the first and second prototypes has a stand-alone device to capture the fingerprint, it is considered as an under or in display fingerprint sensor because it is embedded under the display and gives the user more space for the cell phone display. 

On the other hand, there are many companies that use and develop the stand-alone capacitive fingerprint sensor for cell phone and non-cell phone devices [32] and our proposed solution is based on this approach. The stand-alone capacitive fingerprint sensor is the mainstream of the fingerprint recognition solutions, which is still used by Samsung and many other phone producers due to its maturity and high performance with a low cost and low power consumption [5,6,33]. 

Table 1 shows a comparison between the major methods of fingerprint sensing and its advantages and disadvantages and possibility for embedding under or in-cell phone display. 

In this paper, we present a new architecture for the stand-alone CMOS capacitive fingerprint sensing based on a new cell structure with charge sharing detection and a parallel readout circuit with differential integrators. Its differential integrator circuit provides stable sensitivity even under various finger conditions as well as immunity to various noise. The parallel read out circuit enables simultaneous detection for all sensing lines and presents substantial speed up. The area overhead of the parallel differential integrators and comparators is negligible compared with adding an ADC for each row on chip which will be equal to 16 ADCs (in case of 32 Columns × 16 Rows) as used in conventional structures.

Section 2 shows capacitive fingerprint sensing methods and Section 3 shows the proposed differential sensing technique and the architecture of the proposed stand-alone CMOS capacitive fingerprint sensor cell and its readout circuit. Section 4 shows the simulation and experimental results. The conclusion is given in Section 5.

## 2. Capacitive Fingerprint Sensing Methods

**Figure 1:**
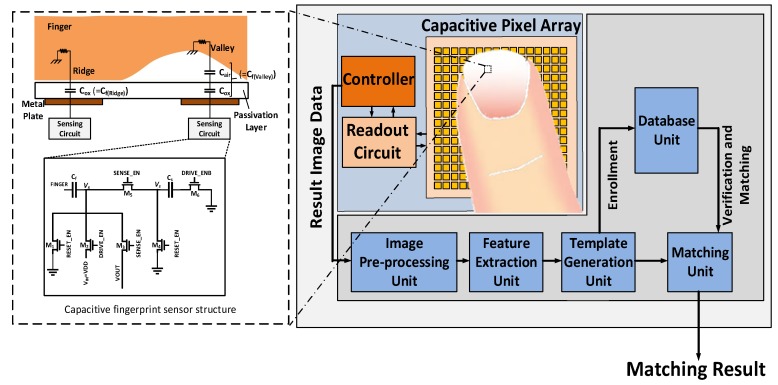
A general scheme of a fingerprint biometric system.

A general architecture of a capacitive fingerprint biometric system is shown in Figure 1. In general, a capacitive fingerprint sensor device includes a capacitive pixel array, a readout circuit and a controller. The capacitive pixel array interfaces and captures the fingerprint data and defines the resolution of the sensor by defining the size and pitch of the cells. The readout circuit converts the capacitance using one of the following approaches: capacitance-to-frequency, capacitance-to-pulse duration, capacitance-to-voltage. Most of the readout circuits contain an analog-to-digital converter (ADC) to convert the sensing values to the digital data, so the controller can conduct post processing on the data. The fingerprint controller controls the driving and sensing circuits for selected sensor cells through the precise timing of control signals. The captured fingerprint biometric data is then fed to the post-processing unit to produce an enhanced image containing the essential information. The feature extraction unit generates the feature vectors, which are either stored in the database or compared with the reference data in the database. If the data is for a new user, then this procedure is called enrollment. However, for verification, the feature vectors of the captured fingerprint are matched with its corresponding reference in the database unit using the matching unit. The matching unit decides if there is a correspondence between inquired fingerprint and the reference [5,33].

**Figure 2:**
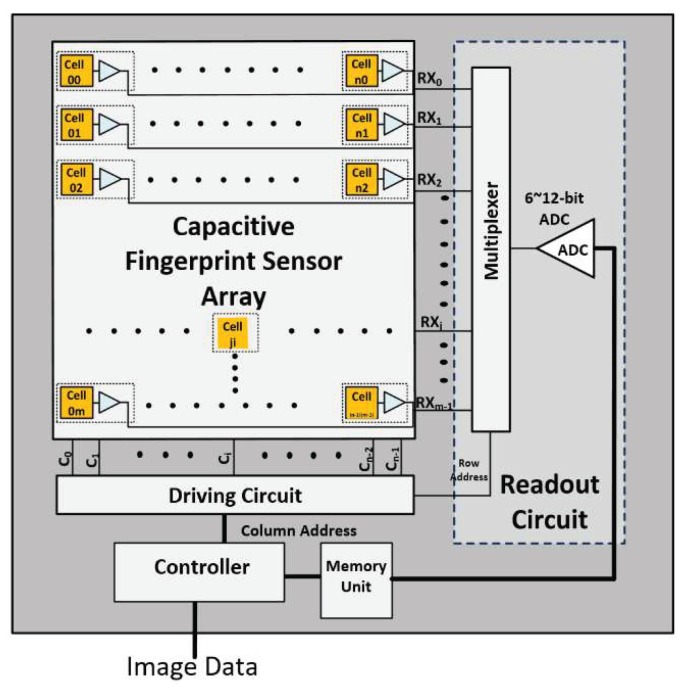
Block diagram of conventional sequential fingerprint sensing architecture.

Most of the capacitive fingerprint sensing methods employ a sequential sensing technique for its simplicity, as shown in Figure 2. These methods apply a drive signal to each cell *Cell_ji_* selected by the column and row addresses [34,35]. In Figure 2, column address *i* selects driving line *C_i_*, so the driving circuit can apply a driving signal to *Cell_ji_*. Then, sensing line *RX_j_* is selected by a multiplexer with row address *j*, and the sensing result of *Cell_ji_* is read out. In most of the sequential sensing methods, the above operation is repeated *n x m* times for *n* rows and m columns to measure one frame of fingerprint patterns [11,12,13,14].

The capacitance changes measured are translated to voltage or current values, and then to digital data via an ADC. The above conventional architectures, however, lack the capability to remove the noise produced from the cells, sensing circuits, or human body. The impact of noise on the detection performance can be substantial unless proper noise cancellation functions are implemented. Common mode noise often imposes large noise signals on the cells and can causes significant loss of sensitivity of the readout circuit. It is, however, hard for the single cell sensing methods to remove the common-mode noise.

To remove the common mode noise effectively, we introduce a differential sensing architecture in this paper. Its differential integrator cancels the common mode noise from every pair of cells. The differential integrator includes an offset compensation technique that is effective in suppressing the input offset caused by process variation and design mismatches [36]. The operation of the differential integrator with input offset compensation and noise cancellation can be found in [36].

In addition, it can eliminate the needs for any threshold voltage that is required in the conventional single cell sensing methods. The proposed architecture identifies ridges and valleys by relative comparison between the two consecutive cells instead of comparing with the threshold. However, a naïve differential sensing method may suffer from the cancellation problem—when the consecutive cells have similar sensing values, the two values cancel each other by the differential sensing circuit [30,31]. Our proposed architecture resolves the cancellation problem by introducing a two-phase detection mechanism with an alternating cell selection.

## 3. Proposed Differential Sensing Technique

### 3.1. Differential Sensing Architecture

**Figure 3:**
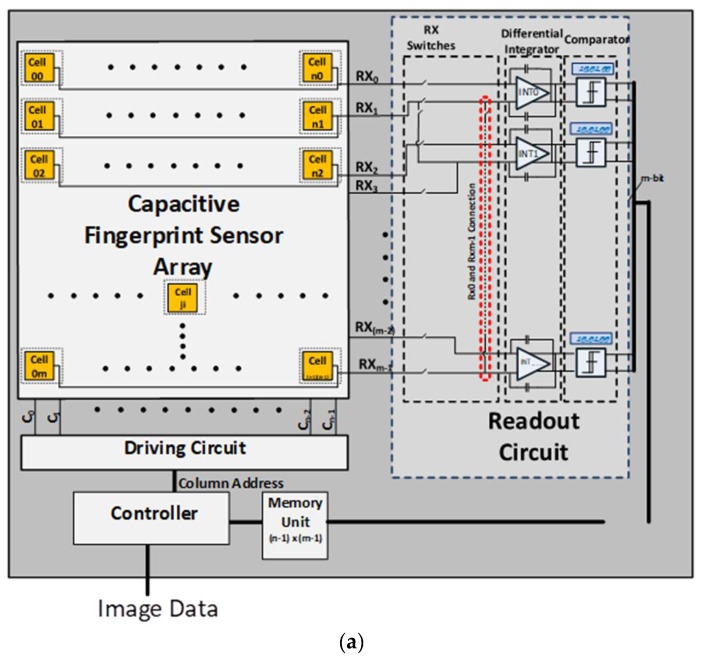

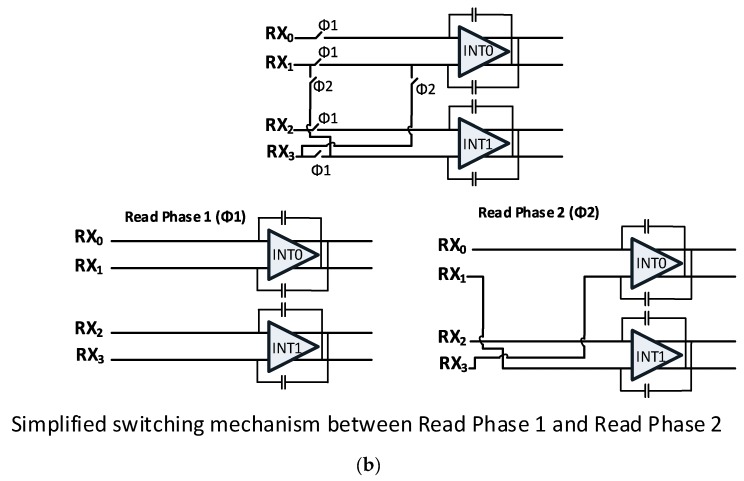
The proposed capacitive fingerprint differential sensing scheme. (**a**) Block diagram; (**b**) simplified switching mechanism between read phase 1 and read phase 2.

The proposed differential sensing architecture for a capacitive fingerprint sensor is shown in Figure 3. The overall architecture consists of a capacitive sensor array, a driving circuit, a parallel read out circuit, and a controller as shown in Figure 3a. The capacitive sensor array is composed of *n x m* cells that interconnected by *n* columns of driving lines (channels) and *m* rows of sensing lines (channels). Each sensor cell employs a charge sharing technique to efficiently measure the capacitance differences between valley and ridge patterns. The driving circuit generates excitation pulse signal and sequentially applies it to one column, say *C**_i_***, selected by the controller. The parallel read out circuit, on the other hand, reads all rows, *RX_j_* (0 ≤j≤m−1). In each read operation, it configures *RX* switches in two alternating read phases as follows (see Figure 3b).

**Read phase 1**: the *RX* switches connect *RX_j_* and *RX_j+_*_1_ (0≤j≤m−1, j=even numbers) to the differential integrator *INT_k_* $\left(0\;\leq k\leq \left(\frac{m}{2}-1\right)\right)$. 

**Read phase 2**: the *RX* switches connect *RX_j_* and *RX_j+_*_1_  (1≤j≤m−2, j=even numbers) to *INT_k_* $\left(0\;\leq k\leq \left(\frac{m}{2}-2\right)\right)$. In read phase 2, when *j = m−*1, *RX_m−_*_1_ and *RX*_0_ are connected to *INT_k_* $\left(k=\left(\frac{m}{2}-1\right)\right)$.

Thus, for a cell array of m rows, half as many integrators, that is, $\left(\frac{m}{2}\right)$ *INT_k_*’s are needed. Although it employs reduced number of integrators, the readout circuit with alternating phases allows us to measure all pairs of consecutive rows. This architecture, therefore, provides an advantage of a smaller size at no sacrifice of sensing accuracy.

The differential integrator converts the capacitance difference of two cells to a differential voltage output. It repeats multiple integrations to further suppress the noise while increasing the differential voltage output. By adjusting the integration repetition count, we can keep the differential voltage output in the range that leads to the best signal-to-noise ratio (SNR).

One drawback of general differential sensing is a signal cancellation problem. When the two cells have the same patterns as Ridge-Ridge or Valley-Valley patterns, the two signals cancel each other by the differential integrator. Hence, the readout circuit cannot detect the correct patterns. Our readout circuit with alternating phases can, however, resolve this problem. For example, suppose that the *RX*_0_*-RX*_1_ pair has a Ridge-Ridge pattern, while *RX*_2_*-RX*_3_ pair has a Valley-Valley pattern. In read phase 1, *INT*_0_ results in common mode voltage (*V_REF_*) by cancelling the same signals of Ridge-Ridge, and *INT*_1_ also gives *V_REF_* by canceling the signals of Valley-Valley. In read phase 2, however, *INT*_0_ takes *RX*_1_*-RX*_2_ and results in a high voltage output by integrating Ridge-Valley. *INT*_1_ would also result in a high voltage, if *RX*_4_ was a Ridge. The proposed read out circuit employs dual comparators for each differential integrator’s outputs. The dual comparators can classify the patterns of two cells into three different cases: (1) the same pattern; (2) valley-ridge pattern; (3) ridge-valley pattern. The proposed architecture, therefore, does not need analog-to-digital converters (ADCs) which often take up large area—another key advantage. Since the comparator’s outputs are digital values, the proposed architecture stores the results of the compactors directly in the data memory for image processing. 

**Figure 4:**
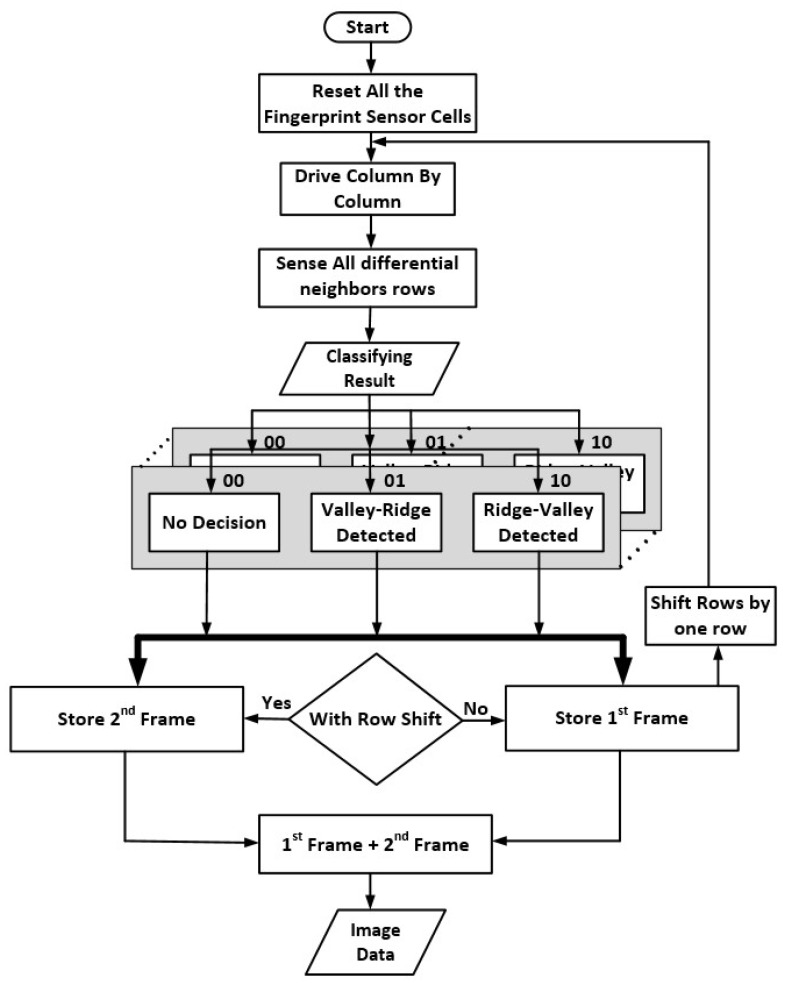
Proposed algorithm to detect the fingerprint features and produce the final image result for further image processing.

Figure 4 shows a flow diagram of the controller’s operation. The controller starts by resetting all the sensors in the array to flush out the previous charge values the cells. Then it selects a column by column sequentially and drives the sensor cell on the selected column. It then reads all the rows (RX lines) concurrently to detect all the cells simultaneously in the selected column.

In read phase 1, the controller measures the differential integrators’ output, and stores the comparator’s results into Frame Memory 1. In read phase 2, it stores the comparator’s results into Frame Memory 2. In both phases, the dual comparator’s results are classified into three values: (1) “00” indicating the same pattern; (2) “01” indicating valley-ridge pattern; (3) “10” indicating ridge-valley pattern. Table 2 shows the output of the comparators for possible fingerprint output cases. Then the post-processing processor compares the two images stored in the two Frame memories. It can identify whether the “00” case was indeed a ridge-ridge or valley-valley pattern. The final image results are sent to the host processor for further image processing and biometric matching algorithm.

### 3.2. Proposed Fingerprint Cell Structure

**Figure 5:**
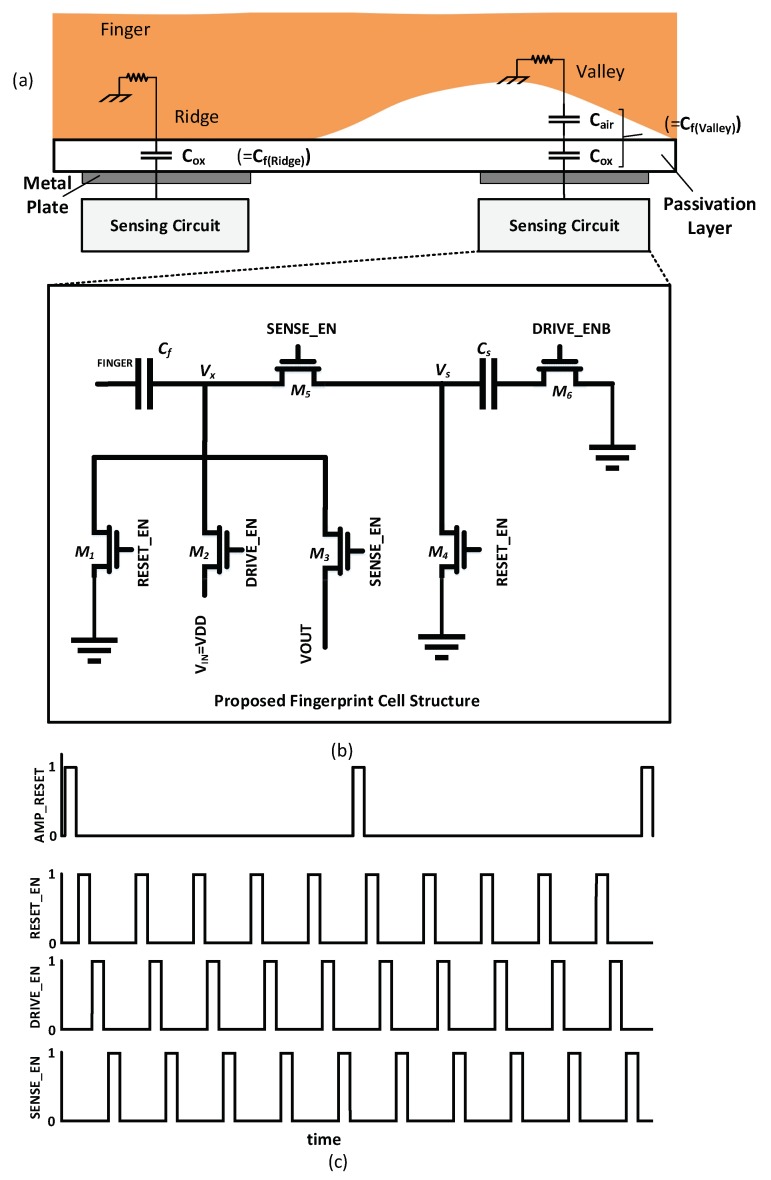
Capacitive fingerprint sensor structure: (**a**) The model of the capacitive fingerprint cell; (**b**) the proposed capacitive fingerprint sensor cell structure; (**c**) the control signal timing diagram.

The proposed cell structure for capacitive sensor is illustrated in Figure 5. The capacitance model formed by the fingerprint and the top metal electrodes of the cell is shown in Figure 5a. The finger skin (Ridge or Valley) represents the upper plate of the finger capacitor *C_f_*. The top metal electrode under the finger skin represents the lower plate of *C_f_*. To model the finger for simulation purposes, we used a ridge capacitance *C_f_* (Ridge) of 44 fF, and a valley capacitance *C_f_* (Valley) of 20 fF. We also used a finger skin resistance *R_f_* of 100 KΩ [11,12]. The capacitance value is inversely proportional to the distance between the two plates. The different capacitance *C_f_* for valley or ridge can be transformed to voltage or current by the readout circuit.

The proposed fingerprint cell structure is shown in Figure 5b. It is insensitive to the parasitic capacitance of the cell by controlling the MOSFET switches. It acts as a parasitic-insensitive integrator [37]. The detection of each sensor cell is conducted by the charge sharing step followed by the charge integration step. During the reset stage when RESET_EN is high, the reference sensing capacitance *C_s_* and the finger capacitance *C_f_* are discharged to the ground.

In the driving mode when DRIVE_EN is high, a driving signal of *V_DD_* level is applied to the finger capacitance *C_f_* and charges it to *V_DD_*. DRIVE_ENB (inverse of DRIVE_EN) turns on switch M6 to discharge during the reset stage, while turning off M6 during the driving mode. During the sensing mode, SENSE_EN turns on M5 and DRIVE_ENB turns on M6, so the charge sharing redistributes *C_f_* charge to *C_s_*. Output voltage VOUT reads out the voltage of *C_f_* after the charge sharing. The voltage of VOUT after the charge redistribution in the sensing mode can be calculated as following:

In the Reset Mode, all capacitors are connected to ground to discharge any residual charge in the cell:

(1) $$Q_{f}≅0,$$

(2) $$\;Q_{s}≅0,$$

In the Driving Mode, the applied signal charges the *C_f_* based on its capacity (Ridge or Valley) through *M*2:

(3) $$Q=Q_{f}=V_{x}\times \;C_{f}=V_{DD}\times \;C_{f},$$

$Q=V_{DD}*\;C_{f}$, where $V_{x}$ is connected to $V_{DD}$ through switch *M2* in this mode. And,

(4) $$Q_{s}=\;V_{s}\times \;C_{s}\;=0,$$

$Q_{s}=0$, where, $V_{s}$ is connected to ground in this mode.

In the Sensing Mode, the accumulated charge on *C_f_* is redistributed between *C_f_* and *C_s_*. A small size is chosen for *C_s_* to increase the dynamic range of VOUT. The optimal size of *C_s_* depends on the estimated size of *C_f_*. For our design, we chose *C_s_* as small as possible to increase the sensitivity to detect small changes in *C_f_*. We estimated *C_f_* as 40–80 fF for ridges and 5–20 fF for valleys considering the measurement data of finger skin capacitance in [11,12,13,14,15,16,17], Refs. [25,26] and the pdk technology used in our experiment. Based on these estimations and based on the minimum possible value defined by the technology we choose *C_s_* of 67 fF. Since *C_f_* and *C_s_* are in parallel connection, the total charge is redistributed between *C_f_* and *C_s_*:

(5) $$Q=V_{x}\times \left(C_{f}+C_{s}\right)=V_{OUT}\times \left(C_{f}+C_{s}\right),$$

where $V_{x}$ is connected to $V_{OUT}$ through switch *M*3 in this mode and the output voltage $V_{OUT}$ is calculated using the charge conservation principles as following:

(6) $$V_{DD}\times \;C_{f}=V_{OUT}\times \left(C_{f}+C_{s}\right),$$

(7) $$V_{OUT}=\frac{C_{f}}{C_{f}+C_{s}}\times \;V_{DD},$$

By measuring the variation of VOUT, the ridge and valley on the fingerprint can be identified. The output voltage VOUT may, however, decrease sharply based on the finger’s skin conditions. To alleviate this problem, our readout circuit employs a differential integrator with adjustable integration steps, which we elaborate in the next sub-section. 

### 3.3. Parallel Readout Circuit Using Differential Integrators

**Figure 6:**
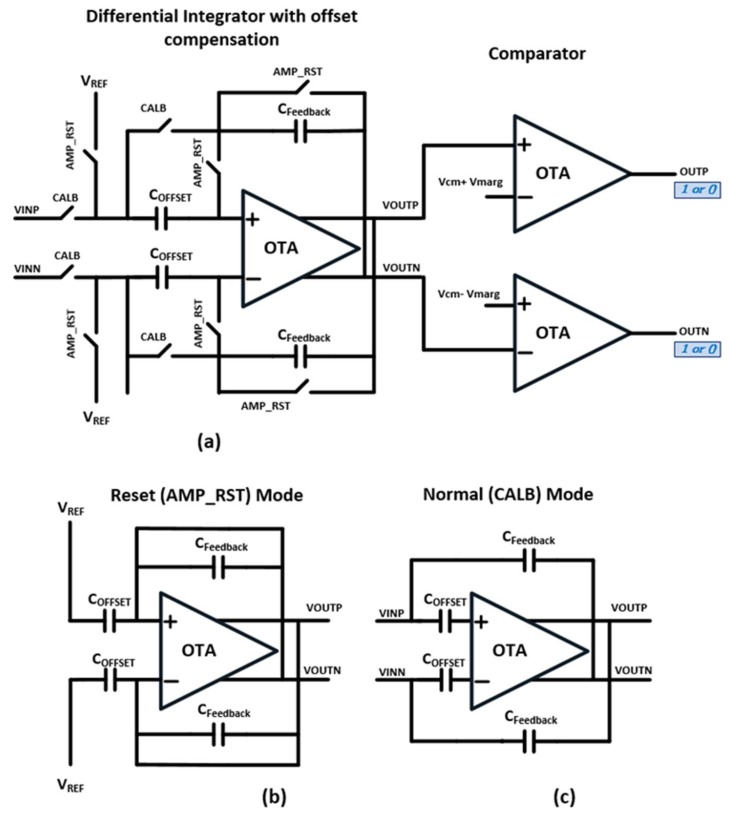
Differential Integrator with offset compensation and Comparator. (**a**) Differential Integrator structure and its configuration in (**b**) Reset Mode and (**c**) Normal Mode.

The overall structure of our parallel read out circuit is highlighted by dotted box in Figure 3. It consists of m/2 differential integrator circuits for a sensor array of m rows. The differential integrator is illustrated in Figure 6. It is composed of a fully-differential operational transconductance amplifier (OTA), feedback capacitors *C_Feedback_*, offset cancellation capacitors *C_OFFSET_*, and control switches AMP_RST and CALB as shown in Figure 6a. The differential operation reduces common-mode noise sources and enhances the signal swing. The integration of charge is performed upon capacitors connected in feedback *C_Feedback_* around a fully-differential amplifier. The resulting total charge is converted to a voltage.

Each integration cycle begins with the reset mode as shown in Figure 6b. It resets the integrator outputs *VOUTP* and *VOUTN* to the common-mode voltage *V_REF_* by turning on switch AMP_RST. The integrator then moves to the normal mode as shown in Figure 6c, where it repeats the integration steps for a specified count. In each integration step, the integrator accumulates the charges redistribution of *C_f_* and *C_s_* to *C_Feedback_* and increases the differential voltage swing between *VOUTP* and *VOUTN*. In each integration step, the controller repeatedly drives and reads the corresponding sensor cell. 

During the sensing mode, the controller turns on switch CALB, so the integrator charges CFeedback. During the drive mode, it turns off CALB, so the integrator can hold its output voltages. As described in Figure 3, each integrator *INT_k_* is shared by two consecutive *RX* lines. If the inputs from the two *RX* lines are the same, for example ridge-ridge or valley-valley combination, then the differential integrator encounters the signal cancellation problem. In other words, the integrator outputs *VOUTP* and *VOUTN* would be closed to the common-mode voltage *V_REF_* of the integrator. Naïve differential sensing methods would not be able to identify whether the input patterns were ridge-ridge or valley-valley.

**Figure 7:**
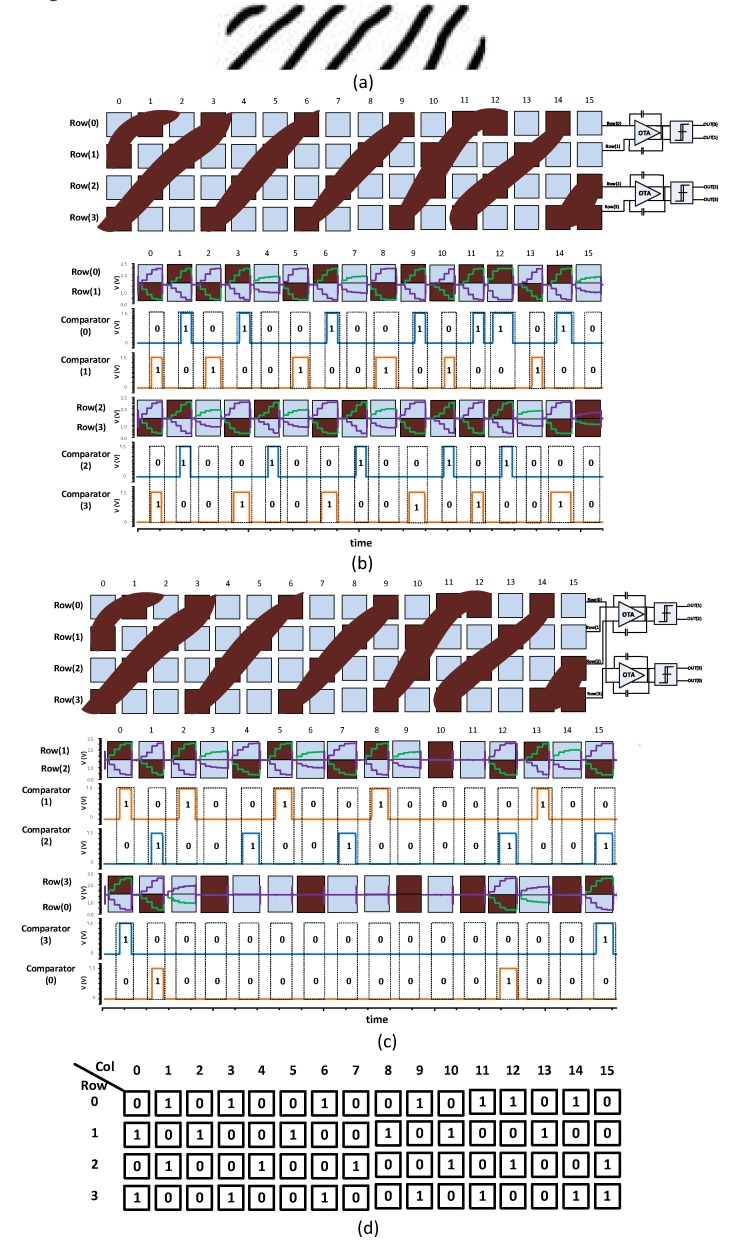
Proposed method to enhance the detection of finger features based on differential readout circuit. (**a**) A sample of finger features; (**b**) reading the modeled sample of finger features in phase 1 and its comparator output; (**c**) reading the modeled sample of finger features in phase 2 and its comparator output; (**d**) the final image output after comparing the two frames.

In the following example, we describe how our technique can resolve such signal cancellation problems. Figure 7 illustrates an example sensor array of 16 × 4 that detects a fingerprint pattern in Figure 7a. As discussed in Section 2, our technique detects all the rows (*RX* lines) concurrently in two phases. Figure 7b shows how Read phase 1 integrates the pairs of *RX* signals and produces comparator decisions 00, 01, or 10. Here integrator *INT*_0_ integrates the difference between *RX*_0_ and *RX*_1_, while *INT*_1_ integrates the difference between *RX*_2_ and *RX*_3_. 

Similarly, Figure 7c shows how Read phase 2 integrates the signals and produces comparator decisions. Here, integrator *INT*_0_ integrates the difference between *RX*_1_ and *RX*_2_, while *INT*_1_ integrates the difference between *RX*_3_ and *RX*_0_. The above operations are repeated for every column, *C*_0_, *C*_1_, *…*, *C*_15_. 

The comparator’s decision values from phase 1 (phase 2) are stored in the Frame memory 1 (Frame Memory 2) for every column. The post processing algorithm compares the decision values of every cell from the two Frame Memories and reconstructs a final fingerprint image as shown in Figure 7d. For example, for column 15, the decision values are *INT*_0_ = 00 and *INT*_1_ = 00 from Frame Memory 1, while the values are *INT*_0_ = 01 and *INT*_1_ = 10 from Frame Memory 2. The values 00, 00 from Frame Memory 1 alone cannot reconstruct the final pattern. The additional values 01, 10 from Frame Memory 2, however, tells that the pattern on *RX*_1_*-RX*_2_ changes from a valley to a ridge followed by the pattern on *RX*_3_*-RX*_0_ changes from the ridge to a valley. In this way, the proposed alternating-phase sensing technique is guaranteed to identify all fingerprint patterns as shown in Figure 7d.

**Figure 8:**
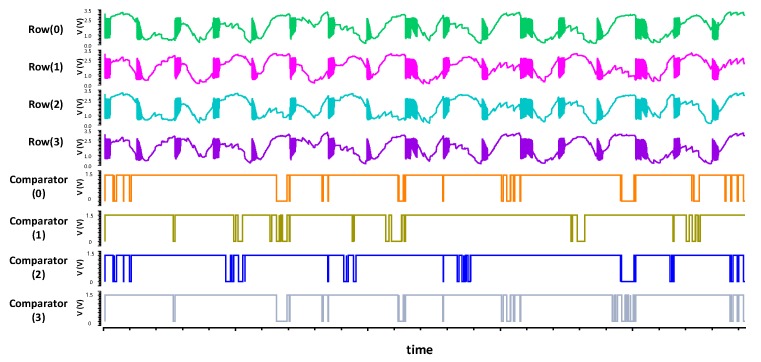
The output of the single-ended integrator.

The simulation of Figure 7 is conducted with random noise added to the sensing signals on all *RX* lines. The simulation results of Figure 7c,d prove that the proposed differential sensing technique can effectively cancel the noise and produce accurate detection results. In contrast, a conventional single-ended sensing method is highly susceptible to even small noise signals. For example, Figure 8 illustrates the sensing results obtained by a single-ended integrator for the fingerprint input of Figure 7a.

## 4. Experimental Results

### 4.1. Implementation of Sensor Chip

**Figure 9:**
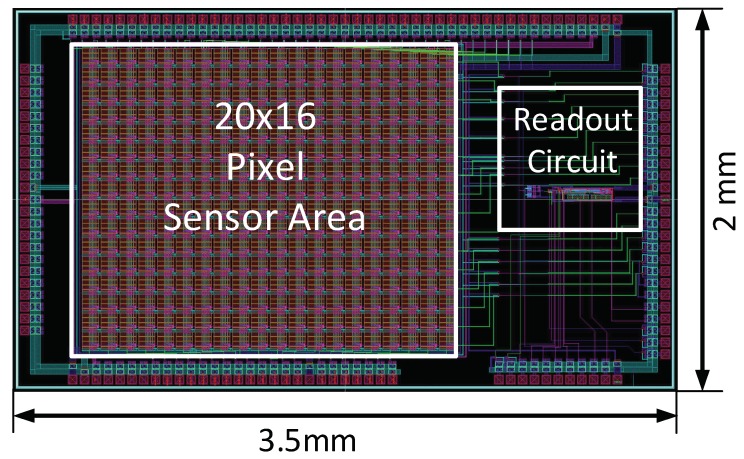
Chip layout.

**Figure 10:**
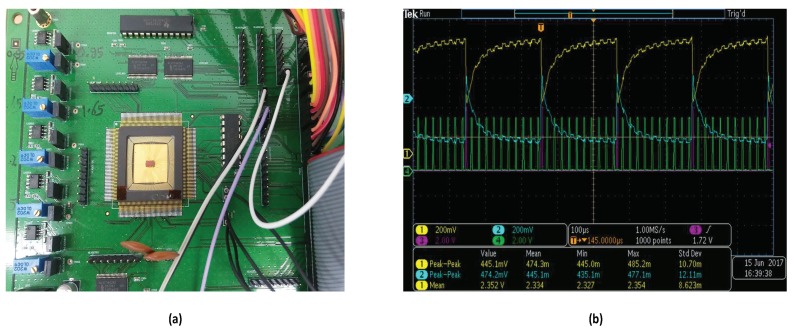
Measurement of the Integrator. (**a**) Test Board of the chip; (**b**) oscilloscope measurement of the integrator output.

We implemented the proposed fingerprint sensing circuit and fabricated a test chip using a 130 nm CMOS process. It consists of a proposed fingerprint sensor array of 20 × 16 sensor cells and a differential readout circuit with alternating read phases. Figure 9 shows the full chip layout design. The silicon area is 3.5 mm × 2 mm including the sensor array occupying 2 mm × 1.6 mm. A typical sensor cell pitch is approximately 40~100 μm, while a common cell size is approximately (50 μm × 50 μm)~(100 μm × 100 μm) [38,39]. The requirements for the Federal Bureau of Investigation (FBI) for fingerprint sensor resolution is higher the 250 dpi [5]. For the current test chip, we chose a cell size of 90 μm × 90 μm and a cell spacing of 10 μm, which gives a resolution of 282.2 dpi, that meets the FBI requirements. Since the proposed cell structure is simple and compact, we can shrink the cell to a much smaller size as well. Table 3 summarizes the specification of the fingerprint test chip and its sensor cell. We measured the test chip using the test board shown in Figure 10a.

The sensing signals measured at the *RX* lines of the sensor array are as small as a few 10’s mV depending on the input patterns. We observed that the proposed differential sensing circuit with 5–10 integration steps can effectively boost the small sensing signals to a large differential voltage of a few volts. For example, with 5 integrations, the differential integrator boosted its differential output to 2.3 V. Figure 10b shows a differential output of the sensing circuits for a ridge-valley pattern with 10 integration steps. Here, the magenta pulses indicate the integrator reset (AMP_RST), while the green pulses denote the sensing enable signals (SENSE_EN). 

### 4.2. Sensitivity Performance Comparison

In general, the performance of capacitive sensing circuits is measured by calculating SNR. We use a similar method of the differential sensing and single-ended sensing techniques.

**Figure 11:**
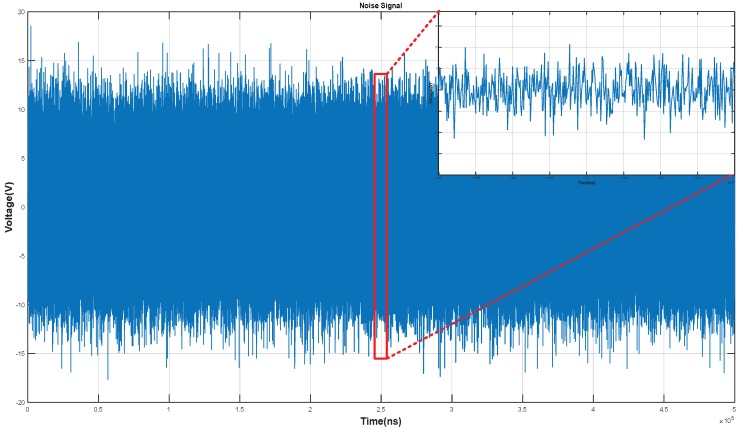
Applied Noise Signal.

Equations (8)–(11) define an SNR that is commonly used for capacitive sensing in the touch screen and biometric sensor industry. To calculate the SNR of the fingerprint sensing circuit, we applied noise signals to the sensor array. Here we used the noise signals that are measured from capacitive sensors. Figure 11 shows such a noise signal. Equation (8) defines the average differential signal voltage for the cases of Ridge-Valley or Valley-Ridge fingerprint patterns. Equation (9) denotes the RMS value of the noise calculated with *n* samples of output signals and noise signals. Equation (10) gives the SNR.

(8) $$Signal_{\left(R\;or\;V\right)}=AVG_{\left(R\;or\;V\right)},$$

(9) $$Noise=\sqrt{\frac{\sum_{i=0}^{n}{\left(Signal{\left(i\right)}_{\left(R\;or\;V\right)}-AVG_{\left(R\;or\;V\right)}\right)}^{2}}{n}},$$

Here, $Signal_{\left(R\;or\;V\right)}$ is the Ridge or Valley signal, $\;AVG_{\left(R\;or\;V\right)}$ is the average of *n* samples for the case of ridge or valley.

(10) $$SNR\left(\mathrm{dB}\right)=20\log\left(\frac{Signal_{\left(R\;or\;V\right)}}{Noise}\right),$$

(11) NR gain (dB)=Output SNR−Input SNR,

Table 4 summarizes three SNR values measured at the input of sensing circuits (i.e., *RX* lines), at the output of a single-ended sensing circuit, and at the output of the proposed differential sensing circuit. The SNR gain defined by Equation (11) provides a fair performance metric regardless of the noise signal amplitude. While the single-ended sensing circuit provides an SNR gain of 13 dB, our proposed differential sensing circuit presents an SNR gain of 53.37 dB—a significant improvement of 40.37 dB with little circuit overhead.

The operation of the proposed scheme depends upon the integrator speed, $f_{INT}$. The differential integrator can operate up to 1 MHz. Though, the required time to read one-column given by the following equation:

(12) $$Scan\;Rate_{\left(One\;Column\right)}=\left(\frac{f_{INT}}{2*N_{Integrations}}\right),$$

Here, $f_{INT}$ is the integrator speed, $N_{Integrations}$ is the number of integration steps. In this case we have integrator speed of 1 MHz and five integration steps. As we read in two phases, so we divide by two. Based on the above estimation for 20 × 16 array, we can calculate the scan rate as:

(13) $$Scan\;Rate_{\left(20\;\times \;16\right)}=\left(\frac{1\mathrm{MHz}}{20*2*N_{Integrations}}\right)=5\;\mathrm{kHz},$$

For a realistic number of a fingerprint array, assuming 128 × 256, we can achieve a scan rate of 780 Hz. This high-speed scan rate helps the software to accurately read the fingerprint features. Table 5 shows the performance comparison with the different fingerprint sensors.

## 5. Conclusions

This paper introduced a differential sensing circuit with alternating read phases aimed at high-speed and high accuracy detection of capacitive fingerprints. We presented a new sensor cell structure with a charge sharing capacitor. The proposed technique can reduce the effect of parasitic capacitances in the cells and overcome the performance loss due to wet or dry finger surface conditions by adjusting the integration step counts. Its differential sensing circuit provides significant enhancement of noise cancellation. In addition, we introduced a sensing architecture using alternating phases, which solves the challenging problem of signal cancellation in differential sensing methods. We implemented the proposed sensing circuit in a test chip with a sensor array of 20 × 16 cells using 0.13 μm CMOS process. Simulations and measurements demonstrated that the proposed differential sensing circuit achieves an SNR gain of 53.37 dB. This is substantial improvement compared to the conventional single-ended sensing circuit that provides only 13 dB.

## Figures and Tables

**Table 1:** Comparison between the major fingerprint sensing methods.

Fingerprint Recognition Technology	Advantages	Disadvantages	Stand-Alone Implementation	Under or In-Display Implementation Status
Capacitive sensing	High-resolution with low cost and low power.	Can malfunction by external electric field and ESD.	Possible, mature, but Needs bazel.	It needs special dealing with the ITO of the touch display. There are many researches in progress to utilize fingerprint sensing.
Optical sensing	Relatively high resolution.	Size, high Cost, easy to spoof.	Lens and prism are required.	In the early stages to be attached under the display. First prototype by Synaptics.
Ultrasonic sensing	3D recognition of fingerprint by ultrasonic distance measurement.	Sweeping method is used for size problem, high cost.	Needs complex manufacturing steps	Needs complex manufacturing steps to be attached under the display. First prototype by Qualcomm.

**Table 2:** The output of the comparators for possible fingerprint patterns.

Fingerprint Pattern	Comparators’ Output
Valley-Ridge	“01”
Ridge-Valley	“10”
Valley-Valley or Ridge-Ridge	“00”

**Table 3:** Characteristics of the fingerprint chip and the sensor cell.

Parameter	Value
Process	0.13 μm CMOS
Array size	20 × 16
Array Area	2 mm × 1.6 mm
Pixel Size (Area)	90 μm × 90 μm
Resolution	282.2
Supply	1.5 V

**Table 4:** SNR for Different Cases of Finger Features.

Comparison Parameter	Readout Circuit Input	Single-Ended Integrator Output	Differential Integrator Output	
Fingerprint Pattern	-	Valley-Ridge or Ridge-Valley	Valley-Ridge	Ridge-Valley
SNR (dB)	0.62	13.62	53.99	54.01
SNR gain (dB)	-	13	53.37	53.39

**Table 5:** Performance summary and comparison with recent fingerprints.

Comparison Parameter	This Work	Refs. [11,40]	Ref. [29]	Ref. [12]	Ref. [10]
Technology (nm)	130	500	180	350	180 + (MEMS) + wafer-bonded
Sensing Method	Capacitive (Stand-alone)	Capacitive (Stand-alone)	Capacitive (transparent)	Capacitive (Stand-alone)	Ultrasonic (Stand-alone)
Sensor Pitch (μm)	100	50	70 × 50	58	~58
Array Size	20 × 16	224 × 256	160 × 192	160 × 192	110 × 56
Sensor IC	Stand-alone	Stand-alone	Stand-alone	Stand-alone	Stand-alone
Fingerprint resolution	282.2	508	500	423	~438
Clock rate (MHz)	1	-	40	40	14
Readout circuit size (mm^2^)	7	64.4	152.4	152.4	~23.7
Frme Rate (Hz)	* 5000	~50	19	-	~380 (Fast Mode)
Supply (V)	1.5	3.3	3.3	3	24
SNR gain (dB)	53.37	-	** 53.3	-	-

* For our prototype we considered 1 MHz clock rate that gives 5 kHz frame rate for 20 × 16 channels. Assuming 128 × 256, we can achieve scan rate of 780 Hz. ** This value for touchscreen.
